# Analyzing Pre-operative Hospital Stay and Incidence of Hospital Acquired Infection: A Retrospective Study

**DOI:** 10.37825/2239-9747.1051

**Published:** 2024-05-09

**Authors:** Maria Costantino, Ornella Piazza, Enrico Coscioni, Emilia A. Vozzella, Walter Longanella, Francesco De Caro

**Affiliations:** a University Hospital “San Giovanni di Dio e Ruggi d’Aragona”, 84121 Salerno, Italy; b Department of Medicine, Surgery and Dentistry “Scuola Medica Salernitana”, University of Salerno, 84081 Baronissi, Italy

**Keywords:** Post-cardiac surgery, Nosocomial infection, HCAIs, Antibiotic resistance, Pre-operative hospital stays, Observational study

## Abstract

**Background and objectives:**

Healthcare-associated infections (HCAIs) pose a significant challenge, impacting patient safety and treatment effectiveness. This retrospective study investigates the correlation between pre-operative hospital stays and HCAIs in ICU cardiac surgery patients.

**Materials and methods:**

Medical records of 35 patients who died post-cardiac surgery in the ICU were analyzed, focusing on the duration of pre-operative hospitalization.

**Results:**

Prolonged pre-operative stays strongly correlate (r = 0.993) with increased HCAIs, indicating a critical risk factor.

**Conclusions:**

The duration of pre-operative hospital stays is pivotal in HCAI risk. Prospective multicenter studies are needed for validation, which is crucial for enhancing patient safety and treatment efficacy.

## 1. Introduction

In 2005, the European Union (EU) recognized access to high-quality healthcare as a fundamental human right, recommending the development of highly reliable and low-risk healthcare organizations where the assessment and management of clinical risk play an important role.

In this context, patient safety has become essential for providing quality healthcare.

Among the issues most frequently compromising patient safety are healthcare-associated infections (HCAIs).

HCAIs are defined as infections contracted during hospitalization that were not clinically evident or in incubation at the time of admission but appear during or after the hospital stay, and are determined by it [1]. This definition includes infections among healthcare setting.

In Italy, the Ministry of Health estimates annually between 450,000 and 700,000 infections in hospitalized individuals [2].

The most common HCAIs involve the respiratory system, urinary tract, bloodstream, and surgical sites. Among the microorganisms frequently isolated in these infections are Gram-negative bacteria such as *Escherichia coli*, *Klebsiella pneumoniae*, and *Pseudomonas aeruginosa*, followed by Gram-positive bacteria like *Staphylococcus aureus*.

In the context of hospital gastrointestinal infections, *Clostridium difficile* represents the most commonly identified pathogen.

An important issue associated with Healthcare-Associated Infections (HCAIs) is the constant increase in antibiotic-resistant bacteria. These bacteria make treatment more challenging, prolong the course of illness and hospital stays, and increase the risk of mortality. Moreover, they can sometimes persist in the body, posing the risk of transmission to other individuals.

The data published by the European Antimicrobial Resistance Surveillance Network (EARS-Net) in 2015 shows that 63.5% of HCAIs have negative economic repercussions (such as loss of workdays and increased healthcare resource utilization). Additionally, HCAIs result in significant human consequences, including the deaths of a considerable number of individuals. These infections are primarily caused by microorganisms resistant to commonly used antibiotics [3–5].

In the Infection Risk Control Program, surveillance and implementation of care measures, as well as hospitalization times, are considered important elements in reducing mortality and infection rates. Surveillance aims to prevent, control, and rapidly detect the onset of infections. Therefore, the World Health Organization (WHO) defines surveillance as a fundamental element, sufficiently comprehensive and accurate, to analyze the distribution and spread of infections and related factors. Numerous European countries have activated continuous surveillance systems in high-risk areas, such as surgical departments and Intensive Care Units (ICUs), to monitor HCAIs and implement corrective measures. Periodically, data are sent to the European Center for Disease Control (ECDC), which publishes the results of such surveillance on its website every year.

In the infection risk control program, hospitalization times are closely monitored alongside surveillance efforts.

In the prevention and control of HCAIs, the number of days of pre-operative hospital stay can be considered a modifiable risk factor [6–8]. Story et al. [6] demonstrated an association between the number of days of pre-operative hospital stay and the development of deep sternal wound infections (DSWI) after cardiac surgery.

An Italian study called KIR-NOS [7,8] was conducted to obtain data on the incidence of Surgical Site Infections (SSIs), including the identification of potential modifiable risk factors for SSIs. Among other findings, the research highlighted that preoperative hospitalization lasting 48 h or more is reported as an independent risk factor for SSIs.

Marchi et al. [9] demonstrated that a pre-operative hospital stays of 2 days or more, as well as emergency surgery, were associated with a higher risk of Surgical Site Infections (SSIs).

Based on the considerations described above and the limited literature data, in our retrospective observational study, we investigated the relationship between the incidence of hospital infections other than DSWIs, such as pneumonia, central venous infection, and severe urinary tract infections, and the duration of pre-operative hospital stay, considering the latter as a risk factor for the development of HCAIs.

## 2. Materials and methods

### 2.1. Study population and study design

The study was designed as a single-center observational retrospective cohort study, according to an a priori defined study protocol.

The Strengthening the Reporting of Observational Studies in Epidemiology (STROBE) guidelines and checklist [10,11] were followed for reporting the whole study.

The analysis focused on deceased patients experienced post-operative severe infections in the Intensive Care Unit (ICU), aiming to assess the extent to which the pre-operative hospital stay contributed to their conditions. Through a retrospective analysis of the medical records, patients who had developed confirmed post-operative infectious complications, as confirmed by microbiological reports, were identified.

Additionally, the type and percentage of presence of the responsible microorganism for the infection were documented through the analysis of infected biological samples.

Surgical site infection samples were not considered for analysis, as there is already extensive literature covering this topic [12–17]. The focus was specifically directed toward post-operative infections occurring in other locations due to the limited existing literature on this specific aspect.

### 2.2. Statistical analysis

For the statistical analysis, patients from the analyzed medical records were stratified into three groups based on the waiting time for elective cardiothoracic surgery (pre-operative hospital stay, expressed in days): ≤2 days, 3–4 days, and ≥5 days. The chi-square test was used for categorical variables, while a logistic regression model was employed to assess the association between the duration of pre-operative hospital stay and the incidence of development of post-operative infections. A significance level of p < 0.05 was considered statistically significant.

## 3. Results

The study population consisted of thirty-five Caucasian deceased patients older than 18 years, from Salerno and its province, with a mean age of 70 ± 8.1 years (range 44–84 years), including 10 females (29%) and 25 males (71%), who had undergone elective cardiac surgery at the "San Giovanni di Dio e Ruggi d’Aragona” University Hospital (U.H. “Ruggi”) in Salerno, Italy, from February 2017 to July 2022, were reviewed.

All patients had sternotomy; forty percent underwent coronary bypass, while sixty percent had valve surgery.

Demographic characteristics of the considered subjects are presented in Table 1.

Among the examined medical records, 40% (n = 14) of the subjects developed post-operative infections confirmed by positive biological samples. The most common infections were pneumonia (93%, n = 13 patients), followed by central venous catheter infections (64%, n = 9 patients), and urinary tract infections (21%, n = 3 patients).

Data analysis revealed a strong positive correlation between prolonged pre-operative hospital stay and a higher incidence of post-operative infections (r = 0.993, p < 0.05). This coefficient indicates a significant and direct association between the duration of pre-operative stay and the occurrence of post-operative infections. This finding underscores the importance of carefully considering the preoperative hospital stay period when assessing the risk of post-operative infections.

Furthermore, the results showed a statistically significant difference in the incidence of infections between patients with a 2-day hospital stay compared to those with 5 or more days (p < 0.05). This discrepancy highlights the importance of optimizing the pre-operative hospital stay period to reduce the risk of post-operative complications (Table 2).

The analysis of microorganisms in blood cultures, broncho aspirate samples, and urine cultures from infected patients (n = 14) highlighted a prevalence of infections caused by gram-negative bacteria (64%, n = 9) compared to gram-positive bacteria (36%, n = 5). *Candida* colonization was detected in 93% (n = 13) of the infected patients, while 21% (n = 3) showed the presence of saprophytic flora.

Microorganisms subject to antimicrobial resistance (AMR) surveillance, such as *K. pneumoniae*, *Enterococcus faecalis*, *P. aeruginosa*, and *Escherichia coli*, were identified.

**Fig. 1:**
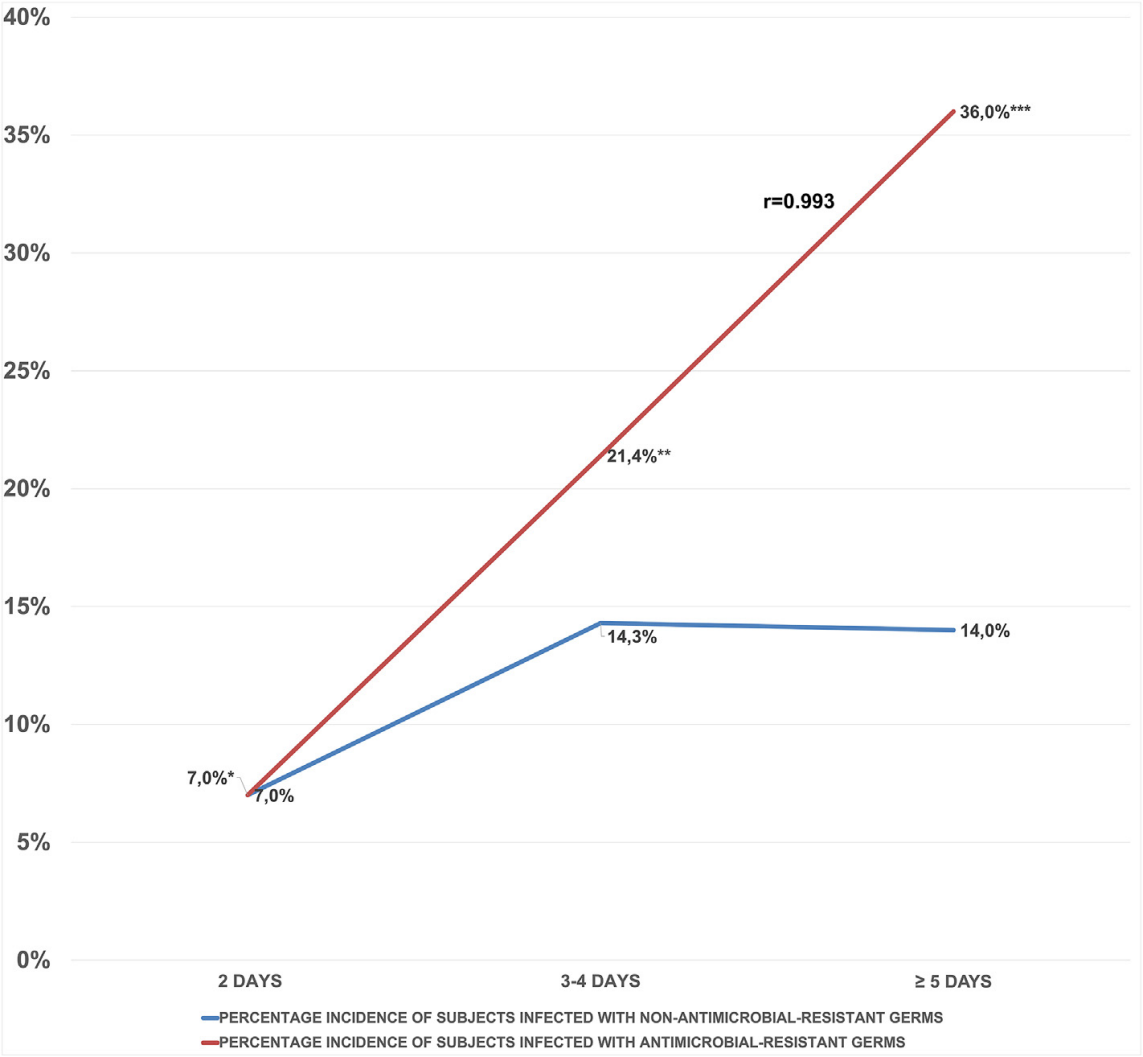
Impact of pre-operative hospital stay duration on infections by antimicrobial-resistant microorganisms: A correlative analysis. * = Pseudomonas aeruginosa. ** = Klebsella pneumoniae - Enterococcus faecalis. *** = Klebsiella pneumoniae - *Klebsiella oxytoca*, a broad-spectrum β-lactamase producer - Pseudomonas aeruginosa - E. Coli.

These microorganisms were selected for their clinical relevance and the need to closely monitor antimicrobial resistance in the hospital environment. Data analysis revealed a significant correlation (r = 0.993) between the duration of pre-operative hospital stay and the incidence of infections caused by these AMR-monitored microorganisms, suggesting an association between prolonged pre-operative hospital stay and an increase in infections caused by antimicrobial-resistant bacteria (Fig. 1).

## 4. Discussion

HCAIs present a critical challenge for patients undergoing cardiac surgery and remain prevalent despite advancements in surgical practices and antimicrobial therapies [18]. Our study exclusively focused on deceased patients who developed infections, excluding cases of Deep Sternal Wound Infection (DSWI) following cardiac surgery procedures.

This focus aimed to address the lack of literature data concerning this specific context. It is well-known that patients undergoing cardiac surgeries commonly face infections such as pneumonia and central venous catheter infections [19]. Therefore, our analysis provides a crucial opportunity to evaluate the effectiveness of existing management protocols and identify potential improvements in handling post-operative infections, with the goal of reducing associated mortality rates, especially among patients with prolonged hospital stays before surgery.

The outcomes of our retrospective observational study have revealed significant associations between the duration of pre-operative hospital stay, the incidence of post-operative infections, and the presence of microorganisms subject to antimicrobial resistance (AMR) surveillance. The strong positive correlation (r = 0.993) discovered between prolonged pre-operative hospital stays and a higher incidence of post-operative infections suggests a potential risk associated with extended hospital stays in the development of these infections. This finding aligns with previous studies [6–9] that have shown an association between pre-operative hospital stay duration and post-operative complications, underscoring the importance of targeted preventive strategies before surgery and optimal management of pre-operative hospitalization.

Notably, there was a marked increase (p < 0.05) in infection incidence among patients with 5 or more days of pre-operative stay compared to those with 2 days, highlighting the importance of monitored preoperative stays.

The prevalent presence of microorganisms under antimicrobial resistance surveillance, such as *K. pneumoniae*, *E. faecalis*, *P. aeruginosa*, and *Escherichia coli*, in patients with longer hospital stays, highlights a potential link between the duration of hospitalization and the onset of infections caused by antimicrobial-resistant bacteria. This revelation suggests a possible impact of hospital stay duration on the incidence of specific infection types, emphasizing the necessity of developing more targeted infection control policies and antimicrobial strategies to address challenges associated with antimicrobial resistance in hospital settings.

The importance of antimicrobial resistance stands out as a critical aspect in our study’s findings. The prevalence of antimicrobial-resistant microorganisms among patients with prolonged hospital stays suggests a potential link between antibiotic resistance and the occurrence of post-operative infections. This highlights the pressing need to understand and tackle antibiotic resistance within HCAIs, emphasizing the necessity for tailored preventive and management strategies aimed at safeguarding antibiotic efficacy and improving clinical outcomes for patients.

However, we acknowledge several limitations in our study. Firstly, the small number of retrospective cases analyzed is inherent to the nature of this investigation. Additionally, our study exclusively focused on cardiac surgery patients, limiting the generalizability of our findings to a broader population. Therefore, larger prospective studies are necessary to confirm and delve deeper into these results.

## 5. Conclusions

Our study explored the correlation between preoperative hospital stay duration, post-operative infection incidence, and pathogen types, offering a preliminary analysis of the prevalence of antimicrobial-resistant germs. Understanding this relationship and its underlying mechanisms could be pivotal in developing more effective preventive and management strategies in cardiac surgery.

The duration of pre-operative hospitalization emerges as a potentially modifiable factor in clinical practice. Optimizing internal resources and enhancing operating room and intensive care unit capabilities could significantly reduce nosocomial infection rates in the sites we considered, curtail excessive antibiotic usage, and consequently lower mortality associated with cardiac surgery hospitalization. This approach holds the promise of substantial long-term improvements for patients.

## Figures and Tables

**Table 1:** Main characteristics of the total study population (n = 35).

Study Population	N = 35
*Age, years*	
mean ± SD	70 ± 8.1
median [range]	72 [44–84]
*Sex, n (%)*	
Male	25 (71)
Female	10 (29)
*Patients with post-operative infections, n (%)*	14 (40)
*Distribution by post-operative infections, n (%)*	
Pneumonia	13 (93)
Urinary tract infections	3 (21)
Central venous catheter infections	9 (64)

**Table 2:** Correlation and Relationship between Pre-operative Hospital Stay Duration and Incidence of Post-operative Infections from the analyzed medical records (n = 35).

Days of pre-operative hospital stay (total patients)	Percentage incidence of post-operative healthcare associated infection (total infected patients)
2 Days (7)	14% (2)
3–4 Days (7)	36% (5)
≥5 Days (21)	50% (7)
Correlation (r)	0.993
χ 2 Test	p-value = 0.190 (2 days vs 3–4 days)
	p-value = 0.043 (2 days vs 5 days or more days)
